# The mechanism of pathogenic α_1_-antitrypsin aggregation in the human liver

**DOI:** 10.1073/pnas.2507535122

**Published:** 2025-11-13

**Authors:** Ibrahim Aldobiyan, Emma L. K. Elliston, Narinder Heyer-Chauhan, Stefan T. Arold, Lingyun Zhao, Brandon Huntington, Sarah M. Lowen, Elena V. Orlova, James A. Irving, David A. Lomas

**Affiliations:** ^a^UCL Respiratory, Division of Medicine and the Institute of Structural and Molecular Biology, University College London, London WC1E 6JF, United Kingdom; ^b^Department of Biochemistry, College of Science, King Saud University, Riyadh 11451, Saudi Arabia; ^c^King Abdullah University of Science and Technology Center of Excellence for Smart Health, Biological and Environmental Science and Engineering Division, King Abdullah University of Science and Technology, Thuwal 23955-6900, Saudi Arabia; ^d^Imaging and Characterization Core Lab, King Abdullah University of Science and Technology, Thuwal 23955-6900, Saudi Arabia; ^e^Institute of Structural and Molecular Biology, School of Natural Sciences, Birkbeck, University of London, London WC1E 7HX, United Kingdom

**Keywords:** serpin, conformational pathology, nonamyloid, ex vivo structural biology, domain swap

## Abstract

Originating 2 to 3 millennia ago in a Scandinavian population, the SERPINA1 Z allele (Glu342Lys) is present in up to 2.5% of populations of Northern European descent and accounts for 95% of severe α_1_-antitrypsin deficiency. The α_1_-antitrypsin Z variant self-assembles into polymer chains that deposit within hepatocytes, predisposing to liver disease. Here, the 4.0Å subunit structure of polymers isolated directly from human liver tissue has been determined using cryoelectron microscopy. Challenges of flexibility, small subunit size, heterogeneous length, and preferred orientations were mitigated using antibody Fab domains and sample preparation strategies. This structure demonstrates that the formation of polymers in vivo involves self-incorporation of an exposed structural element (the reactive center loop) as an additional β-strand into the central β-sheet of α_1_-antitrypsin and displacement of a C-terminal region from one subunit with incorporation into the next. Unlike amyloid aggregation, this well-folded structure partially recapitulates a conformation adopted during normal function of the protein. These perturbations to the constituent α_1_-antitrypsin subunits of human tissue-derived polymers are consistent with a pronounced stability, their tendency toward long-chain forms, the ability of a subset to undergo canonical secretion, and the action of a class of small molecules that block polymerization in vivo.

## Significance

The self-assembly of members of the serpin superfamily into chains of molecules (“polymers”) contributes to the progression of their associated conformational pathologies. Here, the subunit architecture of polymers that form in human tissue has been directly characterized by cryoelectron microscopy (cryo-EM). The subunit reconstruction shows that polymerization involves a misfolding event: The C-terminal region, normally self-incorporated in α_1_‐antitrypsin, is donated to another molecule, and conversely, a loop that is normally exposed is instead self-incorporated. This directly demonstrates the basis on which a nonamyloid domain swap can occur in vivo, as well as the application of cryo-EM to the study of nonideal pathological samples.

α_1_-Antitrypsin is the most abundant circulating protease inhibitor in humans. Approximately 1 g of this protein is synthesized daily by the liver and secreted into the circulation where its primary role is to protect tissues from damage by neutrophil elastase. The SERPINA1 gene that encodes α_1_-antitrypsin is highly polymorphic with a large number of deficiency mutations identified in human populations. The most common severe deficiency allele, and the most actively researched, is the Z variant (rs28929474; Glu342Lys) (1). This mutation induces the formation of ordered polymers that accumulate as inclusions within the endoplasmic reticulum of hepatocytes (2) and circulate as functionally inactive molecules (3, 4). The inclusions are associated with the development of liver disease in neonates and adults, while reduced secretion of the protective monomeric molecule predisposes to early-onset emphysema (5). α_1_-Antitrypsin is a serpin; other members of this family of protease inhibitors are involved in diverse proteolytic pathways critical to human health and are associated with hereditable pathologies including predisposition to thrombosis (antithrombin), angioedema (C1-inhibitor), and dementia (neuroserpin) (6).

Serpins can be found in different conformational states; the native structure is characterized by a central five-stranded β-sheet (denoted β-sheet A) and an exposed “reactive center” loop (RCL) whose sequence defines its target protease specificity (*SI Appendix*, Fig. S1 *A* and *B*). Proteolytic cleavage of the RCL causes it to become embedded as a central sixth strand of the β-sheet A through self-insertion within the molecule (*SI Appendix*, Fig. S1*C*) (7). In certain circumstances, the RCL can also self-insert without cleavage, resulting in adoption of the inactive monomeric “latent” state (8).

Morphologically, α_1_-antitrypsin polymers are flexible, unbranched “beads-on-a string” chains of up to around 20 to 30 subunits. Most have open ends, although some self-terminating circular forms are observed (2, 9). This is consistent with an intermolecular interaction capable of self-propagation. However, these polymers do not form the higher-order structures observed in amyloid fibrils, but rather their hallmarks and solubility are consistent with formation by nonamyloid ordered aggregation.

Historically, different models for the structure of α_1_-antitrypsin polymers have been advanced, based on the characterization of those induced in vitro under destabilizing conditions and from heterologous recombinant expression of mutants (2, 10–12), but not of those arising naturally in vivo. Accordingly, we have performed structural and biochemical characterizations of polymers isolated from liver tissue. Low-resolution maps obtained by negative-stain electron microscopy (EM) showed shapes of dimeric subcomponents within the polymers to be most consistent with a single intermolecular peptide linkage between adjacent subunits (9). More recently, the subunits of these polymers were found to exhibit high-field resonance equivalence by 1D ^1^H NMR to protease-cleaved α_1_-antitrypsin, and ion mobility mass spectrometry with electron capture dissociation revealed a polymer-associated extended monomeric species whose C-terminus and distal RCL were found to be displaced (13, 14).

Here, we present the 4.0Å cryoelectron microscopy (cryo-EM) subunit structure of pathological Z α_1_-antitrypsin polymers isolated from explanted liver tissue. These data provide direct evidence of formation of polymers in the liver that are mediated by an intermolecular interaction in which the C-terminus of one subunit is incorporated into the cognate position in the next subunit. This is a demonstration of an asymmetric domain swap (15) as the basis for nonamyloid pathogenic ordered aggregation in vivo. More generally, this study shows the application of cryo-EM to obtain structural detail from a naturally arising, disease-relevant complex typified by heterogeneity, extensive intersubunit flexibility, and small subunit size.

## Results

### Fab Labeling and Size Fractionation to Improve the Tractability of α_1_-Antitrypsin Polymer Characterization by Cryo-EM.

**Fig. 1.**
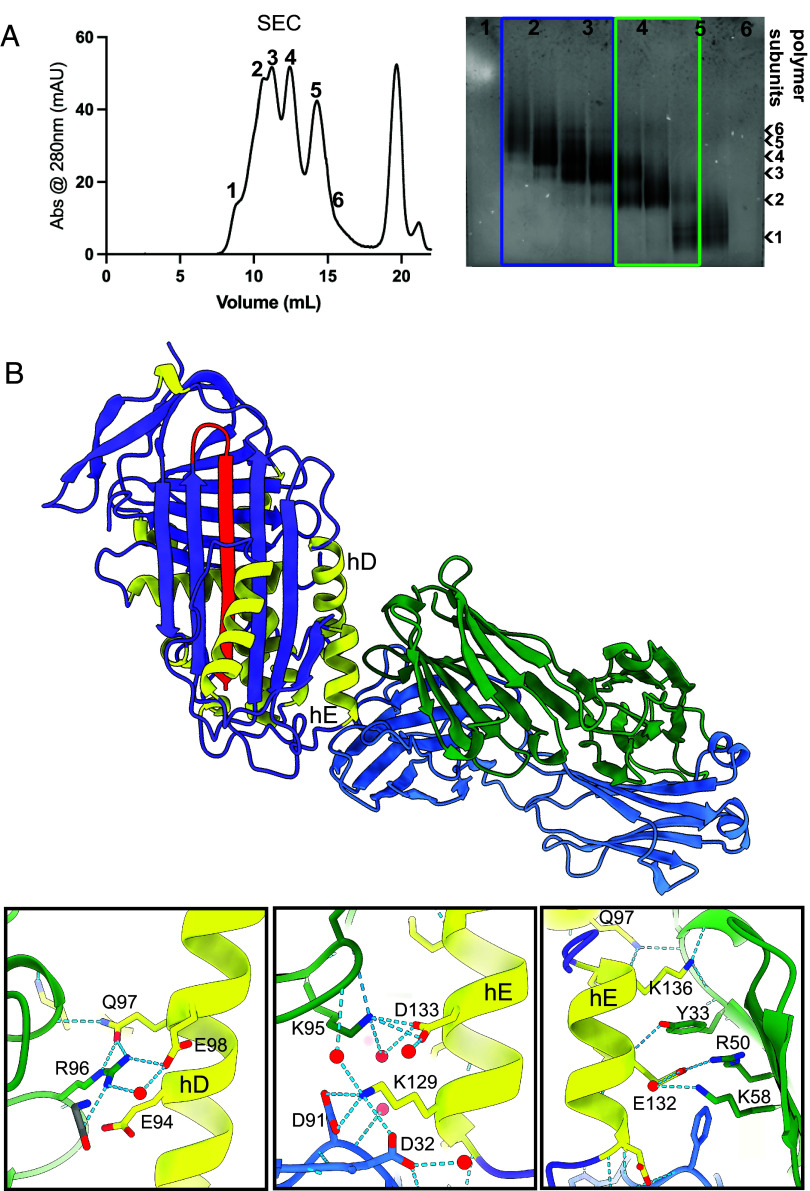
Crystal structure of RCL-cleaved α_1_-antitrypsin in complex with 9C5_Fab_ for comparison with cryo-EM reconstructions. (*A*) Purification of Z α_1_-antitrypsin liver polymers to isolate low and intermediate molecular weight species using size exclusion chromatography (SEC). The chromatogram reporting absorbance at 280 nm (*Left*) and nondenaturing polyacrylamide gel electrophoresis (PAGE) of fractions containing protein to assess their polymeric nature (*Right*) are shown. Fractions corresponding to peaks 2 and 3 (blue box) were pooled together and taken forward for 9C5_Fab_ labeling, representing the ZZ:9C5_Fab_ sample. Fractions corresponding to peaks 4 and 5 (green box) were pooled together and taken forward for 4B12_Fab_ and 9C5_Fab_ labeling, representing the ZZ:4B12_Fab_:9C5_Fab_ sample. The number of subunits comprising the polymer in each fraction is indicated by the black arrows. (*B*, *Upper*) The 2.4 Å crystal structure of RCL-cleaved α_1_-antitrypsin in complex with 9C5_Fab_ reported here [PDB accession 9HUD (16)], with heavy and light chains colored in green and blue, respectively. We also report the 2.2 Å crystal structure of 9C5_Fab_ alone [PDB accession 9GJV (17)] in *SI Appendix*, Table S1. (*Lower*) The hydrogen bonding interactions between helices D and E of α_1_-antitrypsin and the 9C5_Fab_ heavy (green) and light (blue) chains.

α_1_-Antitrypsin polymers were isolated from liver tissue of an individual with a PiZZ α_1_-antitrypsin genotype who had undergone orthotopic liver transplantation for α_1_-antitrypsin deficiency-related cirrhosis (9) (*SI Appendix*, Figs. S2 and S3). Size exclusion chromatography was used to isolate those of intermediate molecular weight, primarily ranging from three (~150 kDa) to six (~300 kDa) subunits (Fig. 1*A*, *blue*). Polymer flexibility and the relatively small and featureless constituent ~50 kDa α_1_-antitrypsin monomer subunits presented a challenging target for cryo-EM structural analysis. This was partially ameliorated by using antibody-derived Fab domains.

We have previously generated monoclonal antibodies (mAb) that recognize different epitopes within the α_1_-antitrypsin polymer, including the 9C5_mAb_ (18, 19). The crystal structures of 9C5_Fab_ alone and in complex with RCL-cleaved α_1_-antitrypsin (referred to here as AAT:9C5_Fab_) were solved (*SI Appendix*, Table S1); the latter localized the epitope to helices D and E of α_1_-antitrypsin (Fig. 1*B*). Fab binding at this peripheral epitope would double the effective molecular weight of the monomer subunits from ~50 kDa to ~100 kDa, confer additional visual features and possibly improve the rigidity of the polymers to assist with the subsequent computational alignment of images (9). The fractionated polymers were incubated with a threefold molar excess of 9C5_Fab_ (herein termed ZZ:9C5_Fab_), and unbound Fab was removed by size exclusion chromatography.

**Fig. 2.**
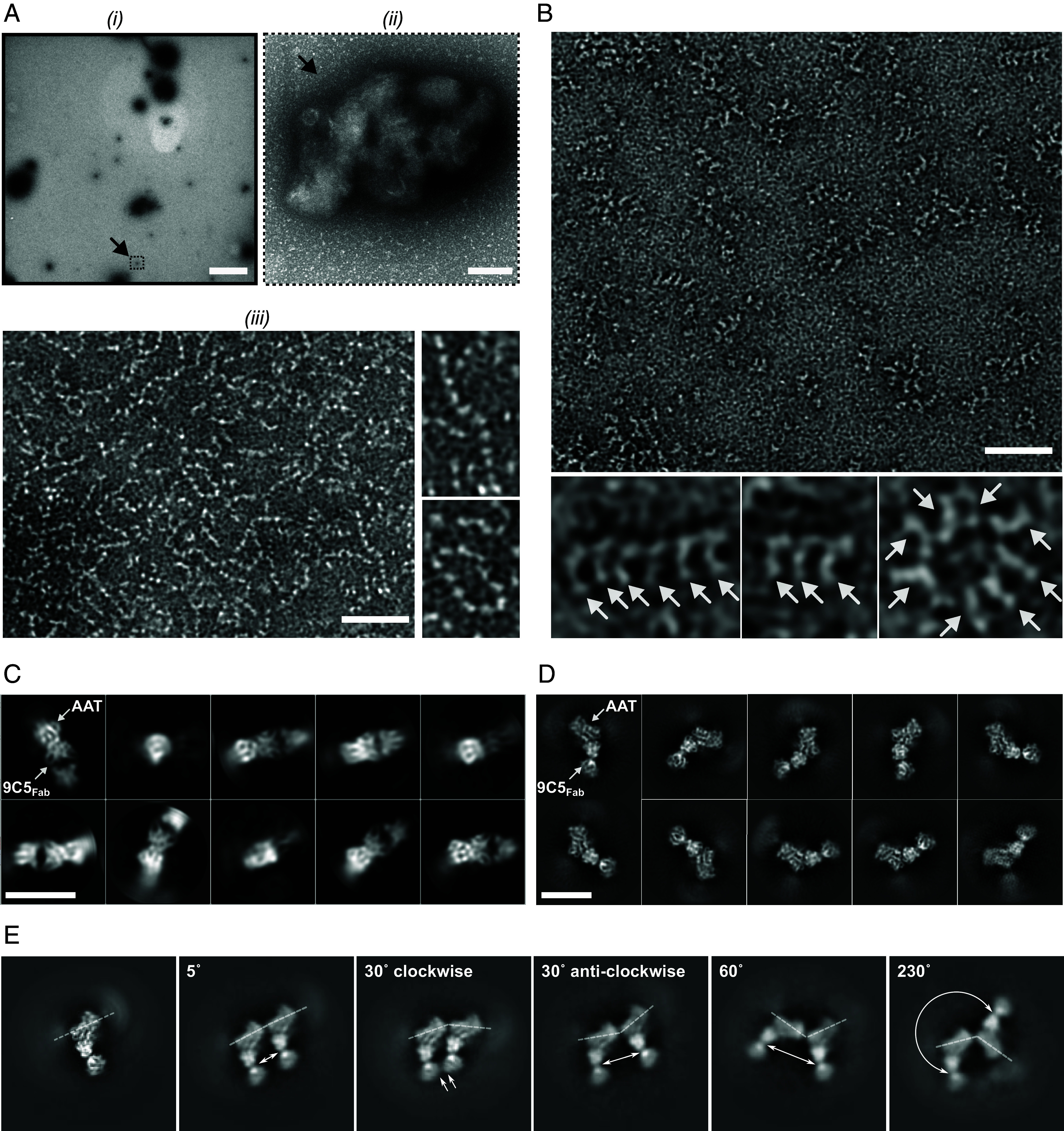
α_1_-Antitrypsin polymers isolated from the human explant liver from an individual with Z α_1_-antitrypsin deficiency. (*A*) Representative micrographs of inclusion bodies extracted from PiZZ α_1_-antitrypsin liver tissue, negatively stained with 2% (w/v) uranyl acetate at (i) 300× (Scale bar, 5 µm) and (ii) 67,000× magnification (Scale bar, 100 nm.) The black arrow in panels (i) and (ii) indicates the same object at different scales. (iii) A negative-stain micrograph of soluble α_1_-antitrypsin polymers released from the inclusions (Scale bar, 50 nm.) Detail at the right highlights chains of α_1_-antitrypsin polymers, both in linear and circular arrangements. (*B*) A representative negative-stain micrograph of these polymers following their fractionation by size exclusion chromatography (Fig. 1*A* and *SI Appendix*, Fig. S3) and decoration with 9C5_Fab_. The 9C5_Fab_ consistently decorates open-ended and circular α_1_-antitrypsin polymers, appearing as distinct protrusions emanating from the polymer chain. A 50 nm scale bar is shown. In the panels, Fab fragments are highlighted by white arrows. The ZZ:9C5_Fab_ polymers were vitrified on grids in the absence (*C*) and presence (*D*) of a graphene oxide support for characterization by cryo-EM. Secondary structural elements can be discerned for α_1_-antitrypsin (AAT) and 9C5_Fab_ components in representative 2D class averages. Scale bars denote 10 nm. (*E*) Examples of the heterogeneity of dimeric subunits and the flexibility of the intermolecular linkage in 2D class averages. The left panel shows a projection of the high-resolution monomeric subunit (described in detail in Fig. 3) by way of reference, in which the dashed line represents the polymer axis. The rotational freedom around the polymer axis in the plane of the micrographs was assessed by measuring the angle between adjacent subunits. The inferred angle is denoted at the top of the 2D classes. The arrows illustrate the distance between the Fab fragments of adjacent subunits.

In comparison to unlabeled polymers extracted from inclusion bodies (Fig. 2*A*), negative-stain EM showed thorough decoration of both linear and circular polymers with 9C5_Fab_ (Fig. 2*B*), visible as protrusions orthogonal to the polymer axis (Fig. 2 *B*, *Inset*).

### Cryo-EM Reconstruction of the Subunit of Polymers Extracted from Liver Tissue.

When plunge-frozen on 300 mesh C-flat grids, the ZZ:9C5_Fab_ sample showed an even distribution of polymers without aggregation within vitreous ice. Twelve thousand micrograph movies were collected at 130,000× magnification (0.93 Å/pixel). The two-dimensional (2D) classes from a manually picked initial particle set of 3,800 Fab-bound polymer subunits revealed density corresponding to both α_1_-antitrypsin and the Fab domains in different orientations. Automated picking using these classes as templates yielded 870 K particles (dataset A). During this process, 2D classes were identified with well-resolved secondary structural elements. However, preferential orientation of the ZZ:9C5_Fab_ subunits also became evident, with the long axis of the α_1_-antitrypsin moieties approximately orthogonal with respect to the plane of the grid. This corresponded with an abundance of end-views of the polymer chain (Fig. 2*C*), in contrast to the predominance of side views in the negative stain images.

**Fig. 3.**
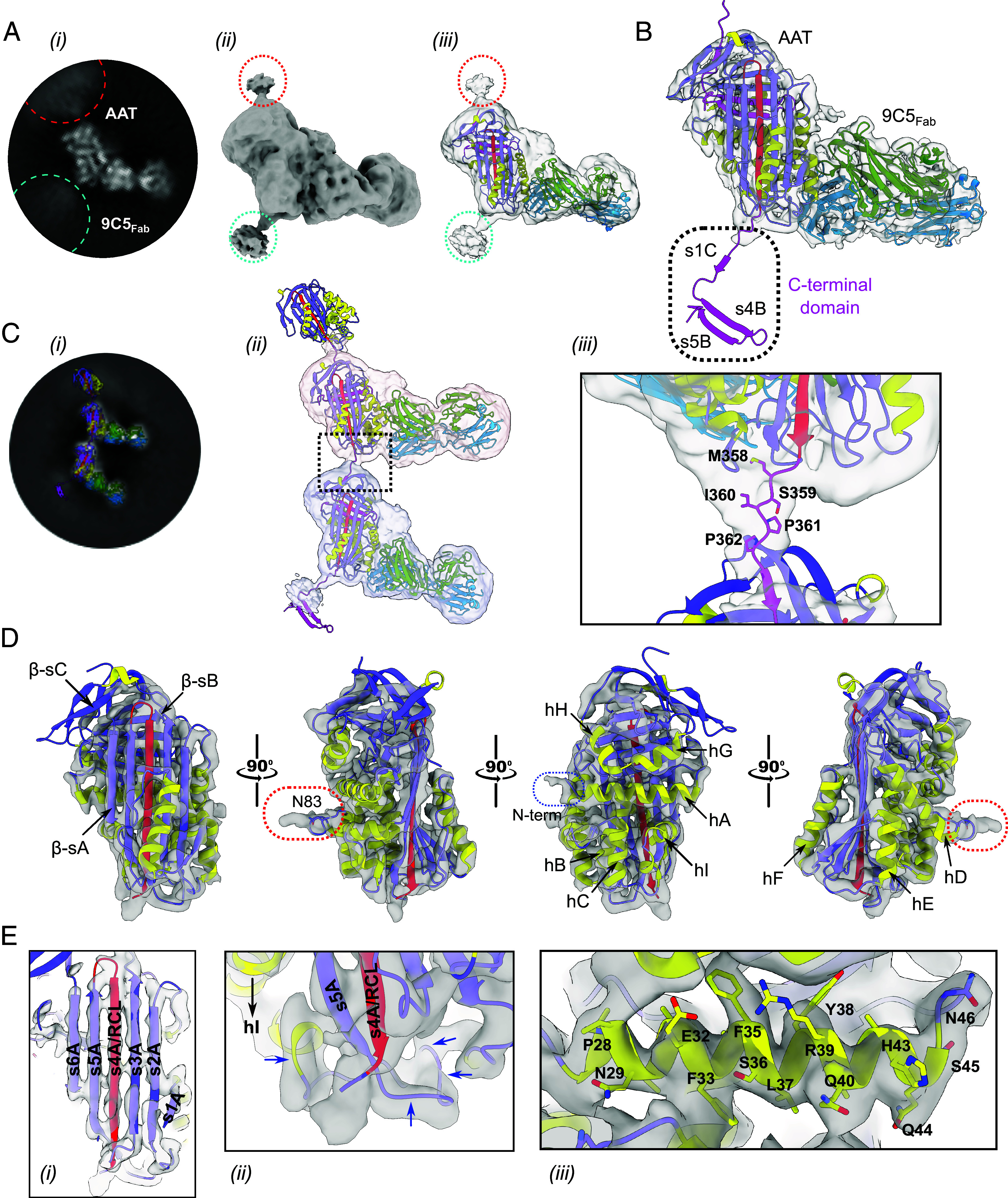
Z α_1_-antitrypsin polymers isolated from the human liver are mediated via a linkage between β-strands 4A and 1C. (*A*) Configuration of the α_1_-antitrypsin polymer subunit (AAT). (i) A representative 2D class average of the monomeric subunit and (ii) a corresponding view of the refined 4.0 Å EM map (EMD-52659) (22) at low threshold levels (contoured at 1.1σ) reveal the additional density of the intermolecular linkage arising from the upper and lower poles of α_1_-antitrypsin (red and cyan circles). (iii) The crystal structure of α_1_-antitrypsin in complex with 9C5_Fab_ corresponds closely to the subunit density (contoured here at 1.1σ), which additionally extends from the top of β-sheet C (β-sC) and the bottom of β-sheet A (β-sA), red and cyan circles, respectively. (*B*) Surface representation of the sharpened 4.0 Å cryo-EM density map of the Z α_1_-antitrypsin polymer subunit in complex with 9C5_Fab_ contoured at 4σ. The crystal structure of an open α_1_-antitrypsin C-terminal polymer (11) subunit, built by reconfiguring PDB entry 9HUD (reported here) (16) with a crystallographic polymer in PDB entry 1D5S (23), was fit into the EM density. α_1_-Antitrypsin is shown in purple, helices in yellow, the RCL in red, and the C-terminal region in magenta; 9C5_Fab_ is shown in green and blue representing the heavy and light chains, respectively. (*C*) (i) A representative 2D class average of a dimer subunit from Fig. 2*E* overlaid with a model of the dimeric subunit shown in (ii) using two monomeric EM density maps, differentially shaded and contoured at 1.1σ. (iii) A close-up of the dashed box shows the intermolecular linkage residues for which density can be observed at threshold levels below ~2σ; residues 358 to 362 correspond to P1-P4’ of the RCL, and the upper-subunit density shown is contoured at 1.1σ. (*D*) The sharpened EM map for Z α_1_-antitrypsin monomeric subunit of the polymer is shown in different orientations at a high threshold level (9.4σ) with respect to the six-stranded β-sheet A conformation [PDB entry 1EZX (24)]. The helices of α_1_-antitrypsin (helices A-I) have been annotated, while additional density for the glycosylation site at residue Asn83 and the N terminus of the protein are marked by the red and blue dashed circles, respectively. (*E*) (i) A close-up view of β-sheet A reveals its six-stranded configuration [PDB entry 1EZX (24)] with self-insertion of the RCL. An intermolecular linkage can be seen as a continuation of the RCL/β-strand 4A density (contoured at 10.5σ) protruding from the lower pole of the protein. (ii) Density (contoured at 9.4σ) for the loop connecting helix I to the base of s5A can be readily discerned (blue arrows). (iii) The degree to which helix A fits the EM density at 6.5σ is shown.

A ZZ:9C5_Fab_ sample was then vitrified in the presence of a graphene oxide support (20), and a dataset of 15,000 micrograph movies at 130,000× magnification (0.65 Å/pixel) was collected. Following automated picking and rounds of 2D classification, approximately 800 K particles were obtained (dataset B). The resultant 2D class averages included resolved secondary structural elements of both α_1_-antitrypsin and 9C5_Fab_, with preferential side-polymer-axis views as had been observed in the negative stain micrographs (Fig. 2*D*). The anisotropy imposed by the graphene oxide supports additionally resolved dimeric subcomponents (Figs. 2 *E* and 3*C*) and diffuse density for adjacent subunits (Figs. 2 *D* and *E* and 3A). A third dataset (C) was obtained without graphene oxide support (at 130,000× magnification, 0.93 Å/pixel) using a sample of predominantly Z dimers and trimers (Fig. 1*A*, *green*) in a ternary complex with both 9C5_Fab_ and 4B12_Fab_ (herein termed ZZ:4B12_Fab_:9C5_Fab_) whose epitopes we found to be nonoverlapping (9, 18, 19, 21) (Fig. 1*B* and *SI Appendix*, Fig. S4 *A* and *B*). Following picking and 2D classification to enrich for target particles, 1,500 K particle images were taken forward for processing, but exhibited more severe anisotropy in their angular distribution, demonstrating a near-exclusivity of end views of the polymer chain. This anisotropy, along with the double-labeling of the polymers with Fabs, produced the effect of pseudomirror symmetry around the central α_1_-antitrypsin molecule (*SI Appendix*, Fig. S4*C*). Subsequent rounds of three-dimensional (3D) classification distributed the particle images among the classes uniformly, with no obvious classes showing improved angular distribution of images. Refinement of a single class containing 600 K particles yielded a map with streaky density along the axis of anisotropy (*SI Appendix*, Fig. S4*D*) and insufficiently resolved features.

**Fig. 4.**
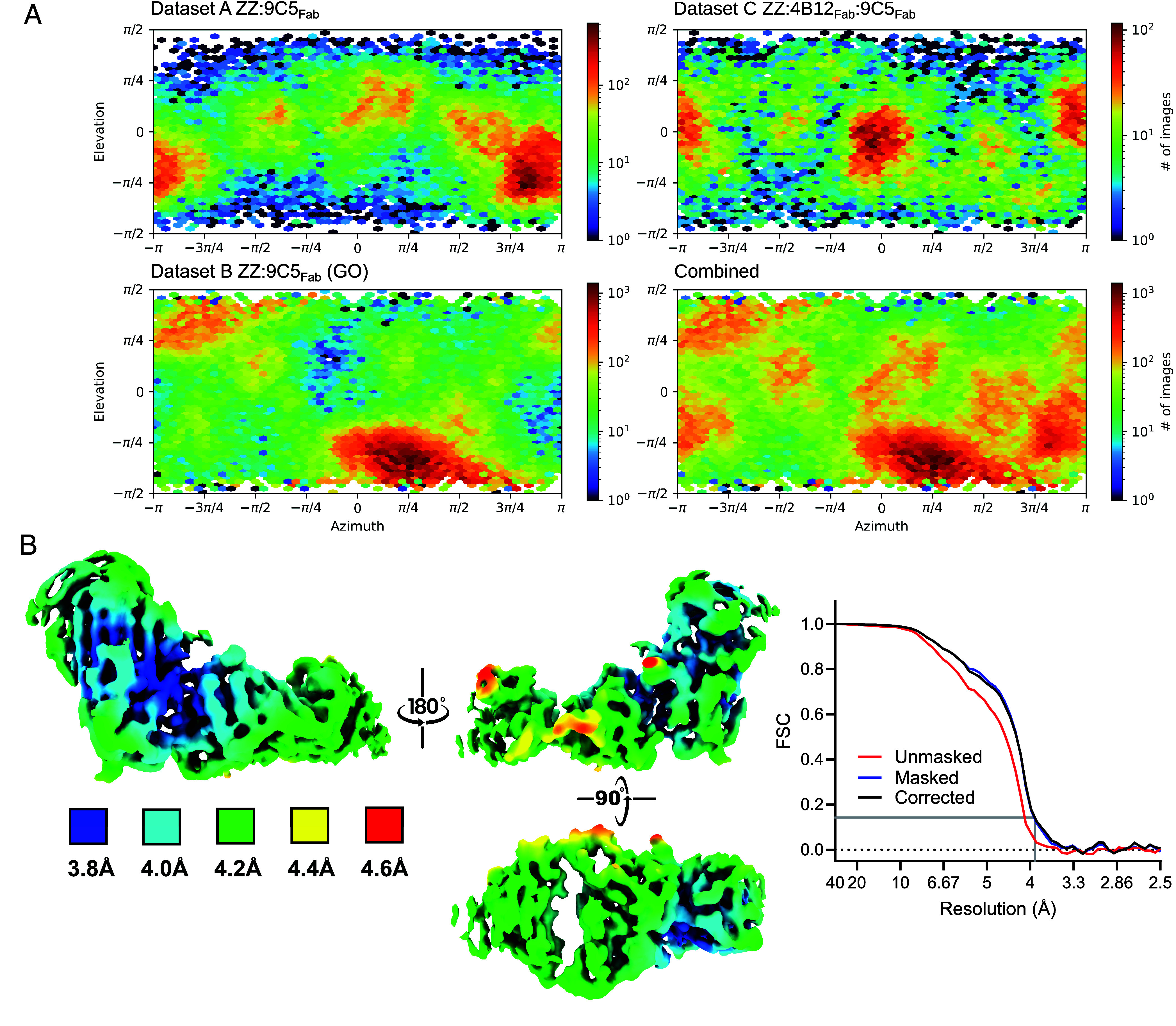
The resolution and angular distribution of the ZZ:9C5_Fab_ subunit reconstruction. (*A*) The orientational distribution of the particles that comprise datasets A, B, and C and the combined data on which the subunit density reconstruction is based. (*B*, *Left*) local resolution estimation of the map, denoted by a color gradient. (*Right*) The Fourier shell correlation (FSC) curves of the final map (gold standard, using a 0.143 cut-off) reported a global resolution of 3.98 Å.

Given the marked anisotropy of the individual datasets, to leverage the complementarity of their preferential particle orientations, particle images from datasets A and B were combined, after rescaling (25) the particle images of dataset B to match the pixel size of dataset A (*SI Appendix*, Figs. S2 and S5, dataset A+B). Classification and refinement of reconstructions produced a map with interpretable structural details at an estimated resolution of 3.9 Å, including densities corresponding to the loops connecting secondary structural elements. The inclusion of ZZ:4B12_Fab_:9C5_Fab_ particle data (the final dataset A+B+C) helped to improve the map quality (*SI Appendix*, Fig. S5). An examination of the distribution of particle orientations for the constituent datasets confirmed that each exhibited a distinct angular anisotropy, which was less pronounced for the combined dataset (Fig. 4*A*).

### Z α_1_-Antitrypsin Forms Polymers in the Liver via a C-Terminal Domain Swap.

This multidataset combinatorial approach revealed the structure of the Z α_1_-antitrypsin polymer subunit in complex with 9C5_Fab_ (Fig. 3 *A* and *B*) at an estimated global 4.0 Å resolution (EMD-52659) (22) and local resolutions between approximately 3.8 Å and 4.6 Å (Fig. 4*B*). Density was observed for all alpha helices (A-I), two of three β-sheets (A and B) and loops connecting these secondary structural elements at higher contour levels (Fig. 3 *D* and *E*), with a less distinct β-sheet C (Fig. 3 *B* and *D*). Comparison with the crystal structure of the AAT:9C5_Fab_ complex, in which α_1_-antitrypsin is in the RCL-self-inserted conformation (Fig. 1*B*), showed close correspondence to this map (Fig. 3 *A*, panel *iii*); a real-space correlation value of 0.79 increased to 0.83 when the Fab C_L_ and C_H_1 regions were permitted to move as a rigid body around the flexible V_L/H_-C_L/H_ linkers (Fig. 3*B*) while a value of 0.82 was obtained for the α_1_-antitrypsin component alone. In particular, density obtained for β-sheet A of α_1_-antitrypsin unambiguously revealed a six-stranded, RCL-inserted configuration (Fig. 3 *E*, panel *i*). The connectivity between β-strand 6A, helix I, and β-strand 5A (Fig. 3 *E*, panel *ii*) was also well-defined, the latter forming a β-hairpin motif with the RCL incorporated as β-strand 4A. Clear density could be seen for the full length of the RCL (residues 341-357) and encompassed Met358 (P1), Ser359 (P1’), Ile360 (P2’), Pro361 (P3’), and Pro362 (P4’) that extended from the lower pole of the polymer subunit to an adjacent molecule (Fig. 3 *B* and *C* panels *ii* and *iii*).

Some 2D class averages of the monomeric subunit exhibited a cloud of density at the top and bottom poles of α_1_-antitrypsin, reflecting the presence of neighboring polymer subunits (Figs. 2 *D* and 3 *A*, panel *i*). Assessment of the subunit density at lower threshold levels revealed a single thread of density extending above β-sheet C (red circle) and below β-sheet A (cyan circle) (Fig. 3 *A*, panels *ii* and *iii*). Subunits representative of the C-terminal polymer (11) (*SI Appendix*, Fig. S1*D*) were fitted into the EM map to produce a model of an α_1_-antitrypsin dimer (Fig. 3*C*). The arrangement of secondary structure elements within the α_1_-antitrypsin subunit, the correspondence with an RCL-self-inserted conformation, and the site of intermolecular linkage density show these liver-derived polymers to be mediated by a C-terminal linkage.

## Discussion

Fibrillar aggregates and complexes such as amyloid, pili, and microtubules involve repeating intersubunit interfaces that generate a symmetry exploitable during processing, facilitating high-resolution cryo-EM reconstructions (26). α_1_-Antitrypsin polymers are known to be flexible (2), to lack a consistent intersubunit surface interface (9), and the corresponding absence of symmetry presents challenges in the context of a subunit size close to the limit of the technique (27). Additionally, with polymer-axis lengths typically an order of magnitude longer than the perpendicular axes, long polymers tend to orient this axis within the XY plane of the images, leading to anisotropy in the resulting angular distribution. To facilitate particle detection and determination of orientational and translational parameters, monoclonal antibody Fab domains with known epitopes were used. Orientational anisotropy was minimized by sample enrichment for different polymer lengths with the combined use of uncoated and graphene oxide-coated grids (*SI Appendix*, Fig. S2).

The resulting data demonstrate a structural basis for polymers that form in the liver of individuals with the α_1_-antitrypsin SERPINA1 Z allele, using material directly isolated from human tissue. The electron density reconstruction shows that these polymer subunits have adopted a low-energy conformation involving self-insertion of the RCL—consistent with the pronounced stability exhibited by polymers with respect to the native conformation (28)—and that their interaction is mediated by the displacement of β-strands 1C, 4B and 5B from one subunit and incorporation into their cognate positions within the next. This is predicated by a C-terminal domain swap (11), whose structural feasibility was demonstrated by X-ray crystallography of a compact self-terminating trimer formed by heating nonglycosylated *Escherichia coli*-derived α_1_-antitrypsin stabilized by an engineered disulfide bond (11). This domain swap involves a single linkage connecting two subunits (Fig. 3 *C*, panels *ii* and *iii*) and is therefore consistent with the considerable flexibility exhibited by polymers (2, 9) (Fig. 2 *A*, panel *iii* and Fig. 2*E*). The labile nature of β-strand 1C (29), at the interface between the two, may be the cause of the reduced local resolution of β-sheet C at ~4.2 Å, compared to the stable core of the molecule in β-sheet A which had an estimated local resolution of 3.8 Å (Fig. 4*B*).

Definition of the Z α_1_-antitrypsin polymerization pathway end-point provides a context for studies that have sought to address the molecular changes that precede it. There is some evidence that polymers form as a consequence of perturbed folding; experiments using in vitro translation (30, 31) and refolding (32) have shown that the presence of the Z mutation introduces a folding delay and population of an alternate conformational state. In mammalian cells expressing Z α_1_-antitrypsin, we have shown the presence of a non-native, nonpolymer species that is recognized by an antibody capable of binding a polymerization intermediate (33). Furthermore, as the native conformation of Z α_1_-antitrypsin shows a stability (33, 34), including at high concentrations for prolonged periods (35), inconsistent with substantial polymerization under physiological conditions, polymer formation would be expected to arise prior to adoption of the native conformation. As such, polymers detectable in the circulation of carriers of the Z allele originate during expression by hepatocytes (3, 33, 36). With demonstration of its central importance as the region that mediates intersubunit interactions in polymers that form in the liver, polymerization therefore likely proceeds during folding from a state in which β-strands 1C, 4B and 5B of the C-terminus have not yet adopted their cognate positions. This contrasts with a hypothesis—based on the “loop-sheet” model that predicates incorporation of the RCL of one subunit by the β-sheet A of the next—that polymer formation proceeds from the natively folded molecule (37).

The C-terminus is, by definition, the final region of a protein to be synthesized. This presents an apparent paradox for wild-type α_1_-antitrypsin: The incorporation of the RCL into the center of β-sheet A is energetically favorable and occurs rapidly upon its release by proteolytic cleavage, and yet at the point of synthesis, the C-terminus to which it is adjacent is not yet anchored to the molecule. A suggestion as to how premature RCL insertion might be averted came from a study which showed, using α_1_-antitrypsin reconstructed from peptide fragments in vitro, that β-sheet A adopts an RCL insertion-competent state only in the presence of a properly situated C-terminus (32). Faster acquisition of native structure by β-strand 1C than by the center of β-sheet A was also observed during refolding of α_1_-antitrypsin following chemical denaturation (38). Thus, two monomeric forms with a displaced C-terminus—an α_1_-antitrypsin intermediate formed during heat-induced polymerization, as well as subunits released from liver-derived polymers—were shown by electron capture dissociation ion mobility mass spectrometry to possess an RCL that was also incompletely self-inserted (13).

Most investigations of α_1_-antitrypsin polymerization have involved in vitro perturbation of natively folded, purified material [Bibr r38][Bibr r39][Bibr r40][Bibr r41](2, 28, 29, 37–43). Extrapolation of these observations to the dynamic, cotranslational folding environment of the endoplasmic reticulum with its attendant chaperones and quality-control mechanisms is nontrivial. It is noteworthy that in a cellular model of disease, more than two-thirds of expressed Z α_1_-antitrypsin has been found to undergo proteasomal degradation (33, 44), and therefore, it cannot be precluded that quality control processes influence the structure of the polymer that ultimately forms.

**Fig. 5.**
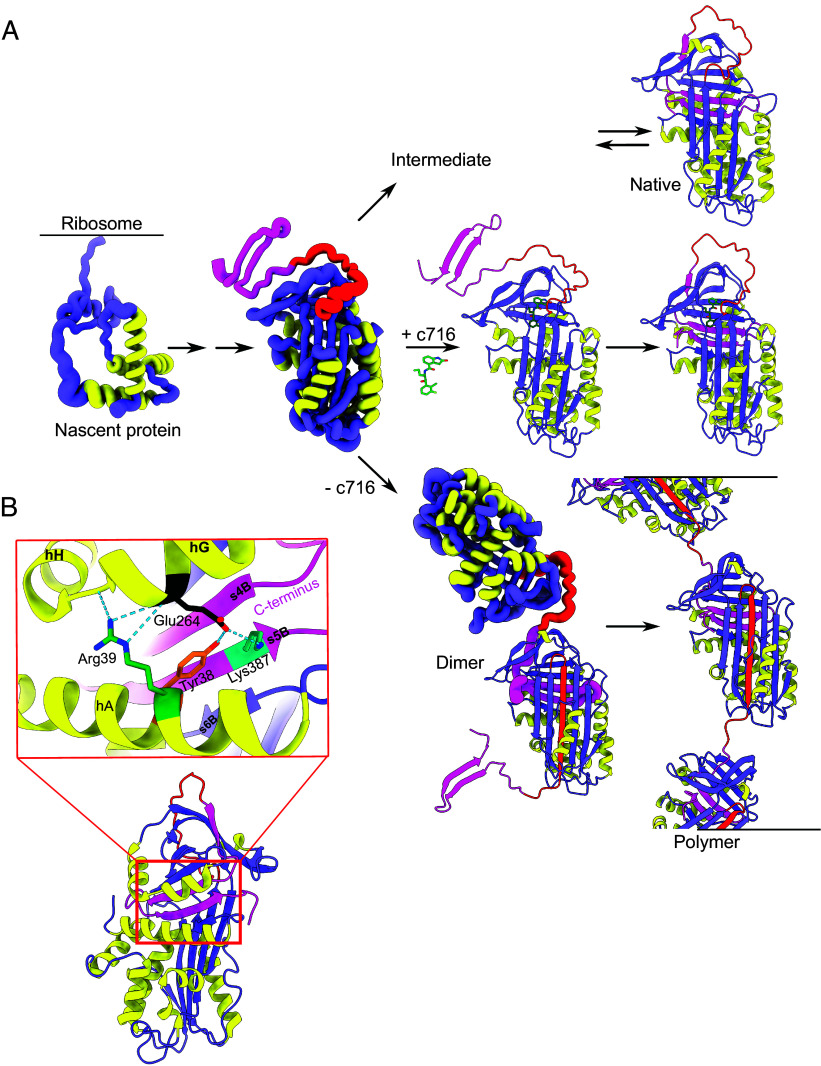
Tentative pathway of Z α_1_-antitrypsin polymerization. (*A*) A possible mechanism, inferred from data discussed in the text, that reflects the ability of a small molecule chaperone, ‘716 (45), to prevent polymerization in the context of a C-terminal asymmetric domain swap model. Characteristics of its binding site suggest that the target ensemble has hallmarks of, or can readily form, the native conformation (35) and is therefore at a late stage of the folding pathway (34, 41), but has not yet incorporated its C-terminus or undergone insertion of its RCL. Throughout the schematic, the RCL is shown in red, and the C-terminal region in magenta. (*B*) The S (Glu264Val, colored in black) and I (Arg39Cys, colored green) variants result in the loss of stabilizing interactions in the trihelical A/G/H region that abuts the C-terminal region. This may explain their ability to copolymerize with the Z variant.

The first demonstration of a structural state that is common to the polymerization pathway in vivo and when induced in vitro was provided by the action of a small molecule inhibitor of polymerization, ‘716. We showed that as well as inhibiting heat-induced polymerization of natively folded protein, this molecule ablates polymer formation and increases the secretion of Z α_1_-antitrypsin in cellular and animal models of disease (33, 45). Methyl-viewed NMR revealed, using purified protein, that the compound binds to and stabilizes an intermediate conformation that is in dynamic equilibrium with the native conformation and substantially populated by the folded Z variant in vitro (35). This state has been observed in purified protein by other techniques to involve increased solvent exposure of the “breach” region and destabilization of the upper portion of β-strand 5A (41, 43) (Fig. 5*A*). The cocrystal structure of ‘716 with recombinant α_1_-antitrypsin correspondingly shows partial perturbation of β-strand 5A and expansion of the breach, with residues from β-sheets A, B, and C contributing to the cryptic pocket (45). Given the ability, at sufficient concentrations of compound, to entirely quench polymerization within the cellular environment with submicromolar IC_50_, binding to the folding α_1_-antitrypsin molecule in vivo might be speculated to involve an ensemble whose native elements are in, or can readily adopt, their cognate positions (Fig. 5*A*). Indeed, the compound both interferes with the transition to the six-stranded RCL-inserted β-sheet A and is unable to bind to α_1_-antitrypsin once this has occurred (13, 45). From the combined data, we hypothesize that the polymerization “decision-point” would occur late in the folding of the molecule but prior to the C-terminal incorporation that determines a native or polymeric outcome.

The structure presented reflects polymerization of a variant that is associated with 95% of cases of severe deficiency of α_1_-antitrypsin (5). It likely also explains some other variants of α_1_-antitrypsin; for example, it is consistent with the observation that the S (Glu264Val) and the I (Arg39Cys) mutants, both of which involve the loss of interactions that stabilize the helix A/G/H trihelical region around the C-terminus, copolymerize with Z α_1_-antitrypsin (4, 42, 46) (Fig. 5*B*). Indeed, we have recently found by evaluation of a panel of pathogenic α_1_-antitrypsin variants expressed in mammalian cells that a number of these are also sensitive to ‘716 treatment and produce polymers well-recognized by the 2C1 antipolymer antibody. However, several were identified to be insensitive to ‘716 treatment and produce polymers poorly recognized by the antibody (47). Further work is required to determine whether these distinct properties in turn reflect differences in polymer structure.

The C-terminal exchange polymerization mechanism is an example of a “three-dimensional domain swap.” A central characteristic of this process is the displacement of a region of one molecule and its incorporation at the cognate site of another molecule, with a “hinge loop” connecting the two (15). For the structure presented here, the RCL residues around 358 to 362, remaining surface-exposed even following RCL insertion into β-sheet A, play the role of this hinge loop. Typically, domain swaps are symmetric and result in closed dimeric or oligomeric complexes, as has been observed for proteins such as cystatin C (48) and RNAse A (49). Those resulting in self-propagating asymmetric “runaway” structures are also known, such as those formed by truncated αA and αB crystallins (50). Both open- and self-terminating domain swaps have been characterized in vitro, generally following induction under destabilizing conditions, but with some attributed to biological function. To our knowledge, no asymmetric domain swap structure has previously been observed in nonamyloid pathogenic aggregates that have formed in tissue.

In conjunction with previous studies of folding and conformational change (9, 11, 30, 31, 33–35, 40, 41, 45, 46), the medium-resolution structure of the Z α_1_-antitrypsin liver-derived polymer subunit explains pathological polymerization within the liver of individuals with Z α_1_-antitrypsin deficiency and provides a basis for further investigation into the pathway by which they form.

## Materials and Methods

### Purification of Proteins.

All chromatography was performed on ÅKTA Pure (Cytiva) or NGC (Biorad) systems. mAb proteins were purified from hybridomas according to published methods (19) and stored in phosphate-buffered saline (PBS) with 0.02% (w/v) NaN_3_. Fab fragments were generated by limited proteolysis using ficin or papain, as appropriate, with commercial kits according to the manufacturer’s instructions (ThermoFisher Scientific) with the subsequent addition of 1 mM E-64 inhibitor.

Recombinant wild-type (C232S) α_1_-antitrypsin was expressed in XL1-Blue *E. coli* cells (Agilent) and purified by affinity chromatography as described previously (28). The RCL-cleaved form of α_1_-antitrypsin was prepared by incubating recombinant α_1_-antitrypsin in a 20:1 molar ratio with Glu-C protease (*Staphylococcus aureus* V8, Sigma-Aldrich) in PBS at 37 °C overnight. Glu-C was then inhibited by adding AEBSF to a final concentration of 1 mM. The extent of cleavage was assessed by sodium dodecyl sulfate polyacrylamide gel electrophoresis (SDS-PAGE). Native noncleaved AAT was removed by heating at 60 °C for 30 min followed by size exclusion chromatography using a Superdex 75 increase 10/300 GL column (GE Healthcare) pre-equilibrated in crystallography buffer [10 mM Tris pH 7.4, 50 mM NaCl, and 0.02% (w/v) NaN_3_].

### Production of the RCL-Cleaved AAT:9C5_Fab_ Complex.

RCL–cleaved recombinant α_1_-antitrypsin was incubated with a threefold molar excess of the 9C5_Fab_ for 3 h at room temperature. The sample was concentrated before the removal of unbound Fab through size exclusion chromatography. The concentration of the complex was determined by measuring the 280 nm absorbance at an extinction coefficient of 9.6 (E 1%). The sample was concentrated using a 30 kDa Amicon centrifugal filter (Millipore) to 10 mg/mL prior to high-throughput screening of crystallization conditions.

### Isolation of Ex Vivo Liver Polymers.

The participant was enrolled in the London Alpha-1 Antitrypsin Deficiency Cohort Study and provided written informed consent. The study was conducted with National Research Ethics Service Committee London—Hampstead approval (REC reference 13/LO/1085). Frozen explant liver tissue (150 g) from a single ZZ homozygous patient was homogenized on ice, then incubated for 90 min at 37 °C at 400 rpm in 300 mL Hanks balanced salt solution (modified with sodium bicarbonate without phenol red, calcium chloride, and magnesium sulfate, Sigma-Aldrich) supplemented with 100 mg of **Clostridium* histolyticum* type IA collagenase (Sigma-Aldrich). Fibrous tissue was removed by filtration through 40 µm pore size cell strainers, the filtrate was centrifuged at 3,000 g for 15 min at 4 °C, and the pellet was resuspended in 25 mL of EM buffer [50 mM Tris pH 7.4, 50 mM NaCl, 5 mM ethylenediaminetetraacetic acid, 0.02% (w/v) NaN_3_] supplemented with 0.25 M sucrose. The sample was dispensed above prechilled EM buffer supplemented with 1.3 M sucrose and centrifuged at 16,000 g for 2 h at 4 °C. The supernatant was discarded, and the pelleted inclusion bodies were washed repeatedly with EM buffer. The soluble α_1_-antitrypsin polymers were released from the inclusion bodies using a Q500 cup horn sonicator (Qsonica) in a water bath prechilled to 6 °C, at 50% amplitude for a total of 6 min using a 15 s on/off cycle. Nonsolubilized material was removed through iterative centrifugation cycles.

The α_1_-antitrypsin polymers were then purified by immobilized metal affinity chromatography (IMAC). They were diluted in IMAC equilibration buffer (50 mM Na_2_HPO_4_ pH7.4, 100 mM NaCl, 30 mM imidazole, 0.02% w/v NaN_3_) and loaded onto a pre-equilibrated copper-charged HiTrap Chelating HP column (Cytiva). Elution was performed over a 0 to 100% gradient of IMAC elution buffer (50 mM Na_2_HPO_4_ pH7.4, 100 mM NaCl, 250 mM imidazole, 0.02% w/v NaN_3_). Elution fractions containing protein were analyzed by SDS-PAGE and nondenaturing PAGE for quality and to identify those containing polymeric α_1_-antitrypsin. The pooled fractions were dialyzed using 10 K MWCO SnakeSkin dialysis tubing (ThermoFisher) overnight at 4 °C into 20 mM Tris pH8.0, purified further using anion exchange chromatography (9), and analyzed by SDS- and nondenaturing PAGE. Polymer-containing fractions were concentrated to 10 mg/mL using a 100 kDa Amicon centrifugal filter (Millipore) and separated by a Superdex 200 Increase 10/300 GL gel filtration column (Cytiva).

### Negative-Stain EM Preparation of Samples and Data Acquisition.

Purified Z α_1_-antitrypsin liver polymers were decorated with a threefold molar excess of the desired Fab (4B12_Fab_, or 9C5_Fab_, or both) and incubated for 3 h at room temperature. Unbound Fabs were removed by size exclusion chromatography. Purified Z α_1_-antitrypsin liver polymers were imaged by negative-stain EM either in the presence or absence of Fab fragments following dilution to 0.03 to 0.05 mg/mL in EM buffer and applied onto glow-discharged copper grids with a continuous carbon film (400 mesh, Electron Microscopy Sciences) as previously described (9). Images of samples were collected using a Tecnai T12 microscope (FEI/ThermoFisher Scientific) operating at 120 keV with a tungsten electron source and a 4 k × 4 k CCD camera (US4000, Gatan). Nominal magnification was 67,000×, which corresponded to a 1.67 Å/pixel sampling at the specimen level. Images were collected with a nominal defocus of −2.0 to −1.0 μm.

### Cryo-EM Specimen Preparation.

Grids were glow discharged (30 mA) using a PELCO Easiglow system (Ted Pella). Graphene oxide deposition was carried out according to ref. 20. After application of 3 μL of sample, grids were incubated for 20 to 30 s in the chamber of a Vitrobot Mark IV (ThermoFisher Scientific, USA) at 4 °C and 94% humidity and vitrified into liquid ethane. For ZZ:9C5_Fab_ and ZZ:4B12_Fab_:9C5_Fab_ without graphene oxide, 400 mesh C-Flat Au R1.2/1.3 (Electron Microscopy Sciences) grids were glow discharged for 30 s and a blot time of 3.5 s and blot force of 0 used; for ZZ:9C5_Fab_ with graphene oxide, 300 mesh UltrAuFoil R1.2/1.3 Holey gold grids (Quantifoil) were glow discharged for 60 s and a blot force of −10 used.

### Cryo-EM Data Acquisition and Processing.

A summary of the cryo-EM data acquisition parameters is provided in *SI Appendix*, Table S2. Preprocessing of the EM data was performed within CryoSPARC (v4.0 and v4.3.1) for ZZ:9C5_Fab_ (dataset B) using Patch motion correction and Patch CTF estimation (51) as well as within RELION (v4.0) for ZZ:4B12_Fab_:9C5_Fab_ (dataset A and C) using MotionCor2 (v1.4 and v1.5) and CTFFIND4 (v4.0 and v4.1) (52–54). The aligned micrographs were inspected and screened for CTF quality to exclude aberration effects or unreliable CTF estimation. For each dataset, between 2 K and 4 K particles were manually picked, ensuring they exhibited varying views of the polymer subunit and bound by the Fab. Following 2D classification, these particles were used as templates for automated particle picking within CryoSPARC. Several rounds of 2D classification were used to remove “junk” particles, ensuring the particle stack contained well-resolved structural features for both the α_1_-antitrypsin and Fab. This was critical in downstream processing as the asymmetric Fab protrusion would improve projection alignment and angular assignment accuracies in downstream processing. The particles were then imported into RELION (v5.0) (55) for 3D classification for downstream processing. Following the refinement of the individual datasets, particles from datasets A, B, and C were combined as described in the text. Processing of the combined datasets was performed in RELION with Blush regularization (55). Throughout 3D classification and refinement, the reference map was low-pass filtered to 20 Å and a soft-edge mask was applied. Sharpening of the map was performed using a B factor value of −150 Å^2^, with a mask of the same dimensions as used in the previous steps. The final resolution of the map was estimated to be 4.0 Å using the gold standard Fourier shell correlation approach with a cutoff value of 0.143. Local resolution estimation was performed within RELION with angular distribution plots generated using CryoSPARC. A detailed flowchart of data processing is presented in *SI Appendix*, Fig. S5.

### Structural Analysis and Visualization.

Cryo-EM density maps and X-ray crystal structures were visualized in ChimeraX (56). Dimer angles in Fig. 2 were measured using Fiji (57). Rigid body fitting within density and stereochemical refinement of linker peptides was performed in Coot (58) and rigid body fitting and real-space correlation calculations in ChimeraX (56).

### X-Ray Crystallography.

For crystallization trials, the samples were buffer-exchanged into crystallography buffer and concentrated to 10 mg/mL. Broad-screen sitting drop approaches against commercially available buffer formulations (Molecular Dimensions and Hampton Research) were performed with 100 nL protein:100 nL buffer drops dispensed using a Mosquito robot (TTP LabTech) and equilibrated against 75 μL of buffer at 20 °C with automatic image acquisition by a Jansi UVEX imaging system (SWISSCI). Mounted crystals were briefly soaked in the respective crystallization buffer supplemented by 10% (v/v) ethylene glycol before plunge-freezing into liquid nitrogen. Data collection was undertaken at the European Synchrotron Radiation Facility (ESRF) ID23-1 and ID30B beamlines. Data reduction, integration, scaling, and merging were performed using XDS (59) and Aimless (60); the structures were solved by molecular replacement using Phaser (61); model refinement was undertaken with Phenix (62); and model visualization and building were performed with Coot (58). The final crystal structures were validated using the online wwPDB validation server (63). The crystal structure of 9C5_Fab_ alone [PDB: 9GJV, 2.2 Å (17)] was solved first and used, with that of Glu-C-cleaved α_1_-antitrypsin [from PDB entry 9GGP (14)], to solve the structure of AAT:9C5_Fab_ [PDB: 9HUD, 2.4 Å (16)].

## Supplementary Material

Appendix 01 (PDF)

## Data Availability

X-ray crystal structures and electron microscopy density reconstructions data have been deposited in PDB and EMDB [9GJV (17), 9HUD (16), and EMD-52659 (22)]. All other data are included in the article and/or *SI Appendix*.
