# Supplementary material for: The mechanism of pathogenic α1-antitrypsin aggregation in the human liver
Source: Proc Natl Acad Sci U S A. 2025 Nov 13;122(46):e2507535122. doi: 10.1073/pnas.2507535122 (PMC12646233; doi:10.1073/pnas.2507535122)
Supplement: Supplementary file 1 — Appendix 01 (PDF) [file pnas.2507535122.sapp.pdf]

Supporting Information: The mechanism of pathogenic α_1-antitrypsin aggregation in human liver

Ibrahim Aldobiyan[a,b], Emma L. K. Elliston[a], Narinder Heyer-Chauhan[a], Stefan T. Arold[c], Lingyun Zhao[d], Brandon Huntington[c], Sarah M. Lowen[a], Elena V. Orlova[e], James A. Irving[a]*, David A. Lomas[a]*

[a] UCL Respiratory, Division of Medicine and the Institute of Structural and Molecular Biology, University College London, London WC1E 6JF, United Kingdom.

[b] Department of Biochemistry, College of Science, King Saud University, P.O. Box 2455, Riyadh 11451, Saudi Arabia.

[c] KAUST Center of Excellence for Smart Health, Biological and Environmental Science and Engineering Division, King Abdullah University of Science and Technology (KAUST), Thuwal 23955-6900, Saudi Arabia.

[d] Imaging and Characterization Core Lab, King Abdullah University of Science and Technology (KAUST), Thuwal, 23955-6900, Saudi Arabia.

[e] Institute of Structural and Molecular Biology, School of Natural Sciences, Birkbeck, University of London, London WC1E 7HX, United Kingdom.

*Joint senior authors

Supporting Tables

Table S1. X-ray data collection and refinement statistics.

	**$9C5_{Fab}$**	**AAT:$9C5_{Fab}$ complex**
PDB accession	9GJV	9HUD
Wavelength (Å)	0.8856	0.9677
Space group	P 21 21 21	P 21 21 2
Cell constants<br>a, b, c, α, β, γ	41.77Å, 58.62Å, 158.84Å<br>90˚, 90˚, 90˚	117.25Å, 239.25Å, 68.94Å<br>90˚, 90˚, 90˚
Resolution (Å)	54.99 – 2.20 (2.32-2.20)	47.65 – 2.42 (2.47-2.42)
Unique reflections	20062 (1721)	74867 (4594)
Completeness (%)	97.40 (99)	99.70
R_{merge}	0.112 (1.802)	0.317 (0.342)
R_{pim}	0.040 (0.619)	0.098 (0.976)
R_{meas}	0.119 (1.913)	0.332 (3.338)
$< I/\sigma(I) >$	10.3 (1.43)	10.18 (1.45)
R_{work}	0.244	0.211
R_{free}	0.262	0.247
R_{free} reflections (%)	1030 (5%)	3839 (5%)
No. atoms	3325	12718
Wilson B-factor (Å^2)	53.6	30.6
Average B, all atoms (Å^2)	73.0	50.0
Anisotropy	0.098	0.202
F_o,F_c correlation	0.93	0.93
R.m.s. deviations		
Bond lengths (Å)	0.25	0.27
Bond angles (°)	0.48	0.48
Ramachandran plot		
Favoured (%)	96	98
Allowed (%)	4	2
Outliers (%)	0	0

Table S2. Cryo-EM data acquisition parameters

	**ZZ:9C5$_{Fab}$**	**ZZ:9C5$_{Fab}$ (GO)**	**ZZ:4B12$_{Fab}$:9C5$_{Fab}$**
Dataset	A	B	C
Microscope	Titan Krios G4 (ThermoFisher Scientific)	Titan Krios D3771 (ThermoFisher Scientific)	Titan Krios G4 (ThermoFisher Scientific)
Facility	Imaging and Characterization Core Lab, KAUST	Electron Microscopy Laboratory, Birkbeck College	Imaging and Characterization Core Lab, KAUST
Accelerating voltage (keV)	300 (CFEG)	300 (X-FEG)	300 (CFEG)
Energy filter (slit width 20eV)	Selectris X (ThermoFisher)	BioQuantum (Gatan)	Selectris X (ThermoFisher)
Detector	Falcon 4i DED (ThermoFisher)	K3 DED (Gatan)	Falcon 4i DED (ThermoFisher)
Magnification	130,000×	130,000×	130,000×
Pixel size (Å)	0.93	0.65	0.93
Exposure (e^-/Å^2)	49.81	43.55	49.94
Defocus (μm)	−2.7 to −1.7	−2.7 to −1.5	−2.7 to −1.7
Micrographs	11968	15366	10395
Grids imaged	2	2	2
Map resolution (Å)	4.4	4.2	4.0
FSC threshold	0.143		
Particle images, final	273961		
Combined map resolution (Å), final	3.98		
Map resolution range (Å)	3.8 - 4.6		

Supporting Figures

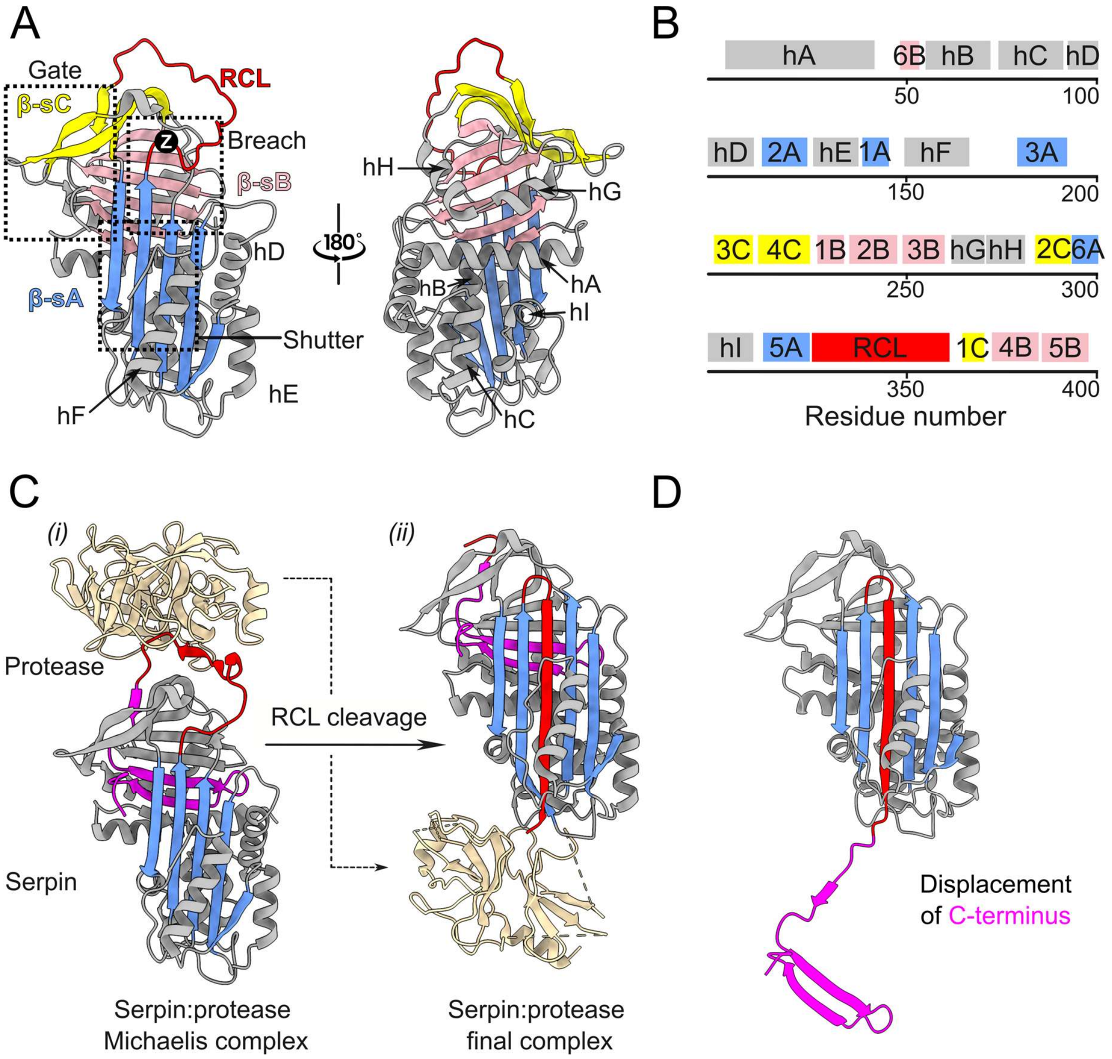

Figure S1. α₁-Antitrypsin structure and function. (A) The structure of α₁-antitrypsin in its native conformation (PDB: 1QLP) with the central β sheet-A (β-sA) in blue, β-sB in pink and β-sC in yellow. The reactive center loop (RCL) is shown in red and helices (hA-I) are indicated by arrows. The breach, shutter and gate regions are highlighted by dashed boxes. (B) The organization of α₁-antitrypsin secondary structural elements is shown in relation to the residue number, using the same color scheme as in panel A, with number-letter designations denoting β-strands and h-letter denoting helices. (C) The suicide substrate mechanism of serpins: (i) The non-covalent serpin:protease Michaelis complex (PDB: 1OPH (1)) forms when the protease (shown in wheat color) docks onto a specific site on the RCL of the serpin (grey); (ii) Proteolytic cleavage of the RCL causes a large conformational change which results in the self-insertion of the RCL within β-sA, with translocation of the protease from the top of the serpin to the lower pole, disrupting the protease catalytic triad and resulting in an irreversible covalent complex (PDB: 1EZX (2)). (D) The structure of the C-terminal polymer subunit (3) involves an inserted RCL in common with protease-cleaved α₁-antitrypsin (C – panel (ii)). The displaced C-terminus is shown in magenta. This subunit of a non-self-terminating polymer was built by reconfiguring PDB entry 9HUD, reported here, with the cleavage-induced crystallographic polymer in PDB entry 1D5S (4). The structures in this and subsequent figures were rendered using ChimeraX (5).

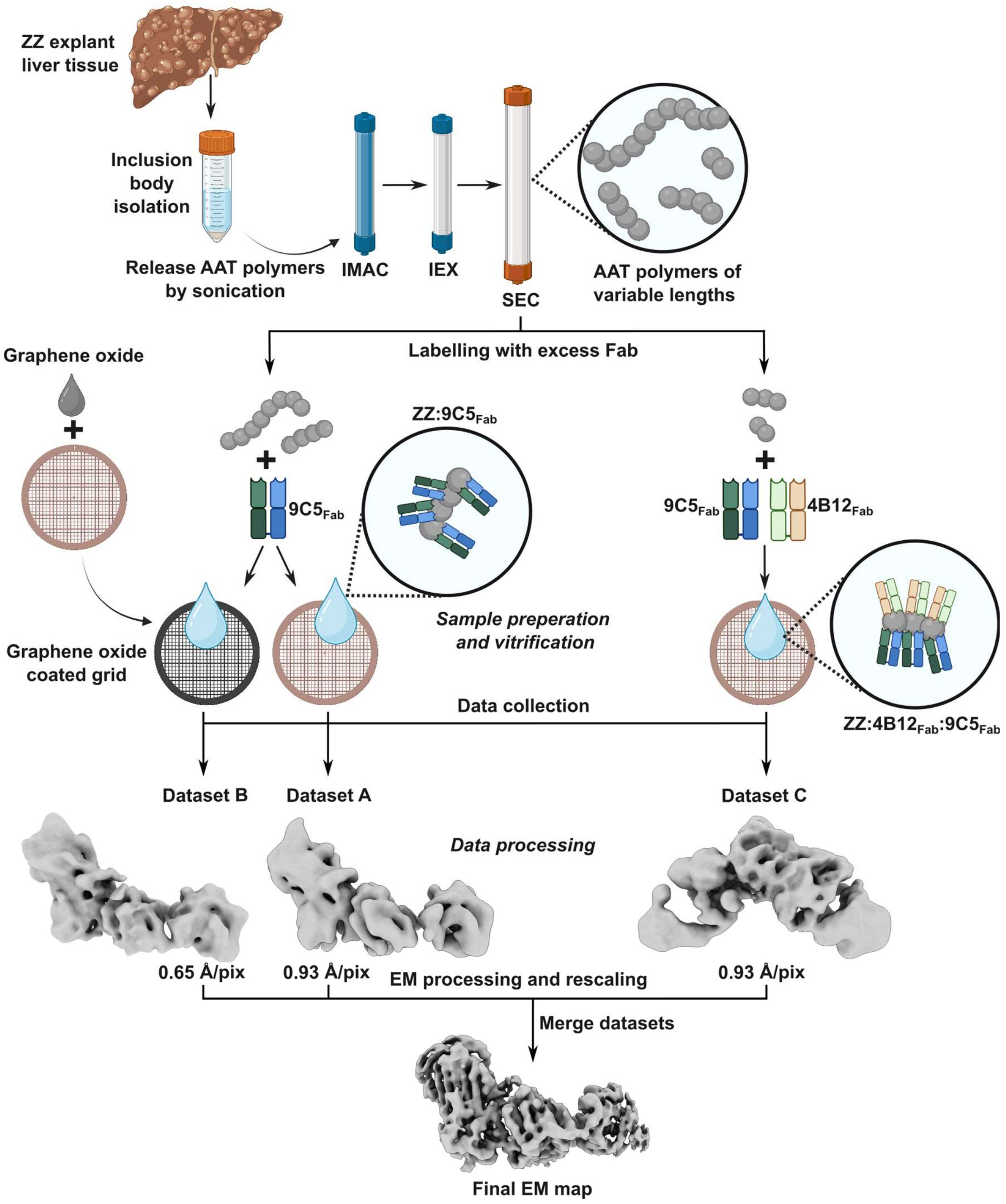

Figure S2. Overview of the project workflow. Explant liver tissue from an individual homozygous for the Z allele (PiZZ) was processed to isolate inclusion bodies containing Z α_1-antitrypsin polymers. Sonication was performed to release the polymers, and they were purified to remove contaminants, with size exclusion chromatography used to further fractionate them based on size. Depending on the size of the polymers, they were either labelled with $9C5_{Fab}$ ($ZZ{:}9C5_{Fab}$) or double-labelled with both $9C5_{Fab}$ and $4B12_{Fab}$ ($ZZ{:}4B12_{Fab}$: $9C5_{Fab}$). The samples were vitrified on grids in the presence and absence of graphene oxide coating and imaged. Each dataset was processed and refined individually. Particle images of dataset B were rescaled to correspond with the pixel size of datasets A and B, before merging the data to produce the final reconstruction. Some elements were produced using BioRender.

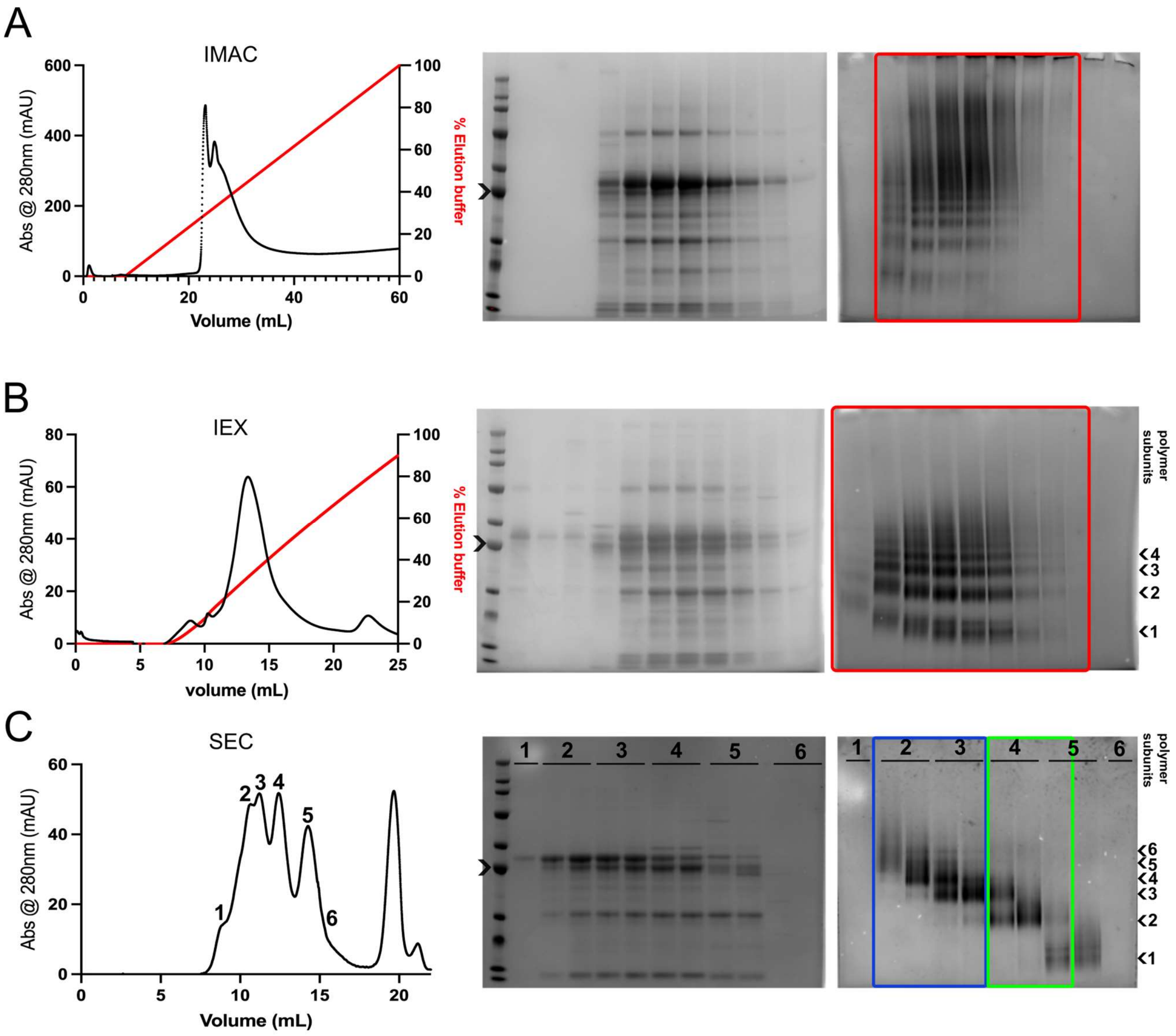

Figure S3. Purification of Z α$_1$-antitrypsin liver polymers to isolate low and intermediate molecular weight species. After sonication of the inclusion bodies to release soluble polymers, polymers were purified sequentially by (A) a copper chelating column (IMAC) followed by (B) an anion exchange (IEX) step and finally (C) size exclusion chromatography (SEC), as summarized in Figure S2. The chromatograms of each purification are shown on the left, fractions containing protein were assessed for purity and polymeric nature by SDS- and non-denaturing PAGE analysis (central and right panels, respectively). Fractions that were taken for further purification are highlighted by the red boxes. For the purposes of cryo-EM, fractions corresponding to peaks 2 and 3 (blue box) were pooled together and taken forward for 9C5$_{Fab}$ labelling, representing the ZZ:9C5$_{Fab}$ sample. Fractions corresponding to peaks 4 and 5 (green box) were pooled together and taken forward for 4B12$_{Fab}$/9C5$_{Fab}$ labelling, representing the ZZ:4B12$_{Fab}$:9C5$_{Fab}$ sample. The black chevron indicates the 50 kDa molecular weight marker in the SDS-PAGE gels. The left and right panels in (C) are also shown in Fig. 1A.

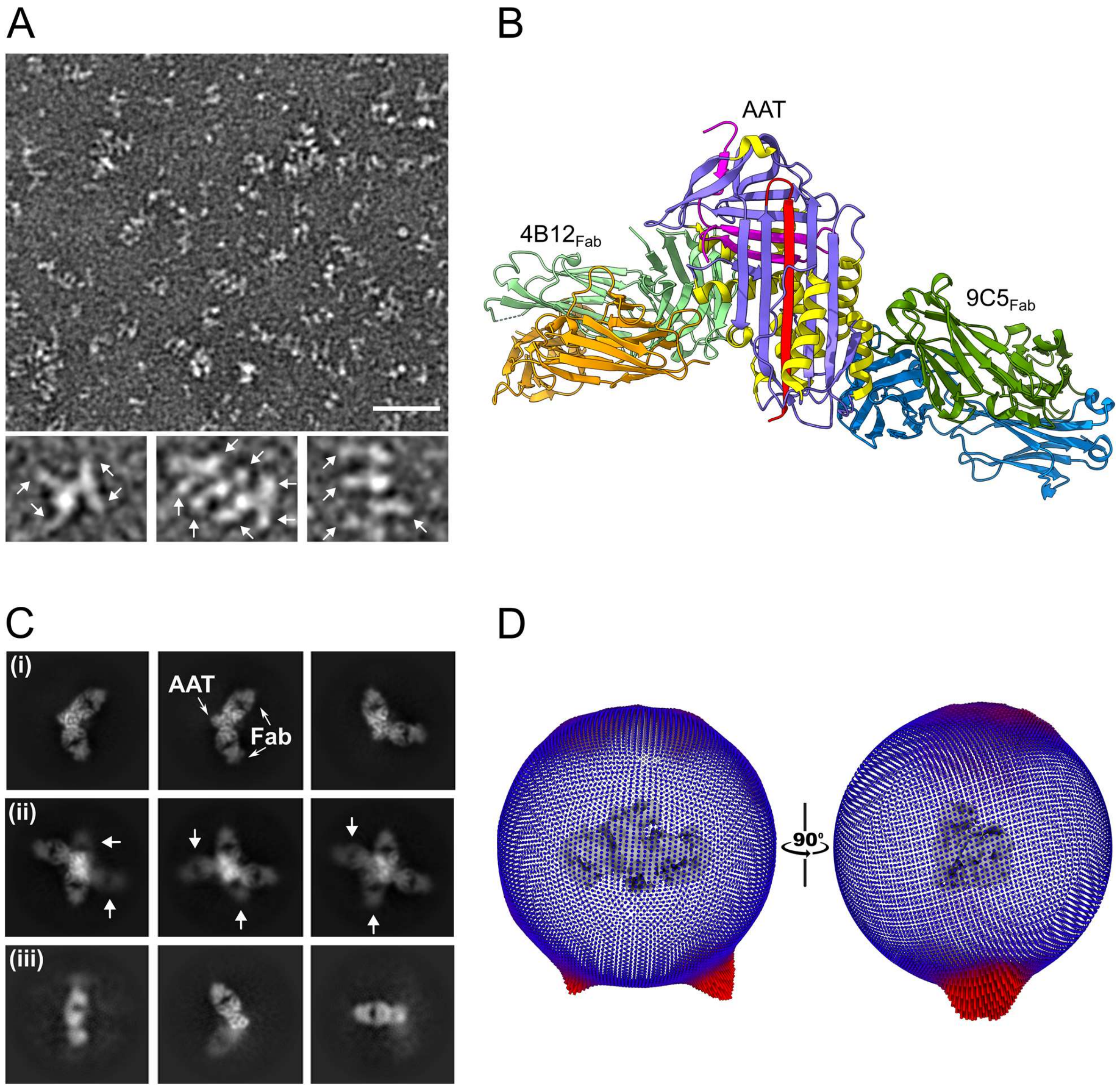

Figure S4. The ZZ:4B12$_{Fab}$:9C5$_{Fab}$ dataset. (A) Under negative-stain imaging, the ZZ:4B12$_{Fab}$:9C5$_{Fab}$ polymers were predominantly dimeric and tetrameric in length, exhibiting both dual and single labelling by Fab fragments as is highlighted by the inset images. Scale bar 50nm. (B) A model of the ZZ:4B12$_{Fab}$:9C5$_{Fab}$ complex, based on the crystal structures of the AAT:9C5$_{Fab}$ (PDB: 9HUD, described here) and 4B12$_{Fab}$ (PDB: 6QU9 (6)), the latter made using previously elucidated epitope sites (6, 7). (C) (i) Following cryo-EM data collection, the 2D class averages of ZZ:4B12$_{Fab}$:9C5$_{Fab}$ particles showed preferred orientation of the end views of the polymer chain resulting in pseudo-mirror symmetry. (ii) Several 2D classes indicated the presence of dimers of ZZ:4B12$_{Fab}$:9C5$_{Fab}$. In such classes, presenting end views of short polymers, the neighboring α_1-antitrypsin subunit is occluded from view, but its decorating Fab fragments are visible leading to the class averages resembling propellers (indicated by the white arrows). (iii) Single-labelled polymer subunits were present among the 2D classes. (D) The angular distribution plot (8) of the ZZ:4B12$_{Fab}$:9C5$_{Fab}$ reconstruction illustrates the anisotropic distribution of the particle views. The height and color of the cylinders are proportional to the number of particles (from blue to red) in these orientations. In the figure, 'AAT' denotes α_1-antitrypsin.

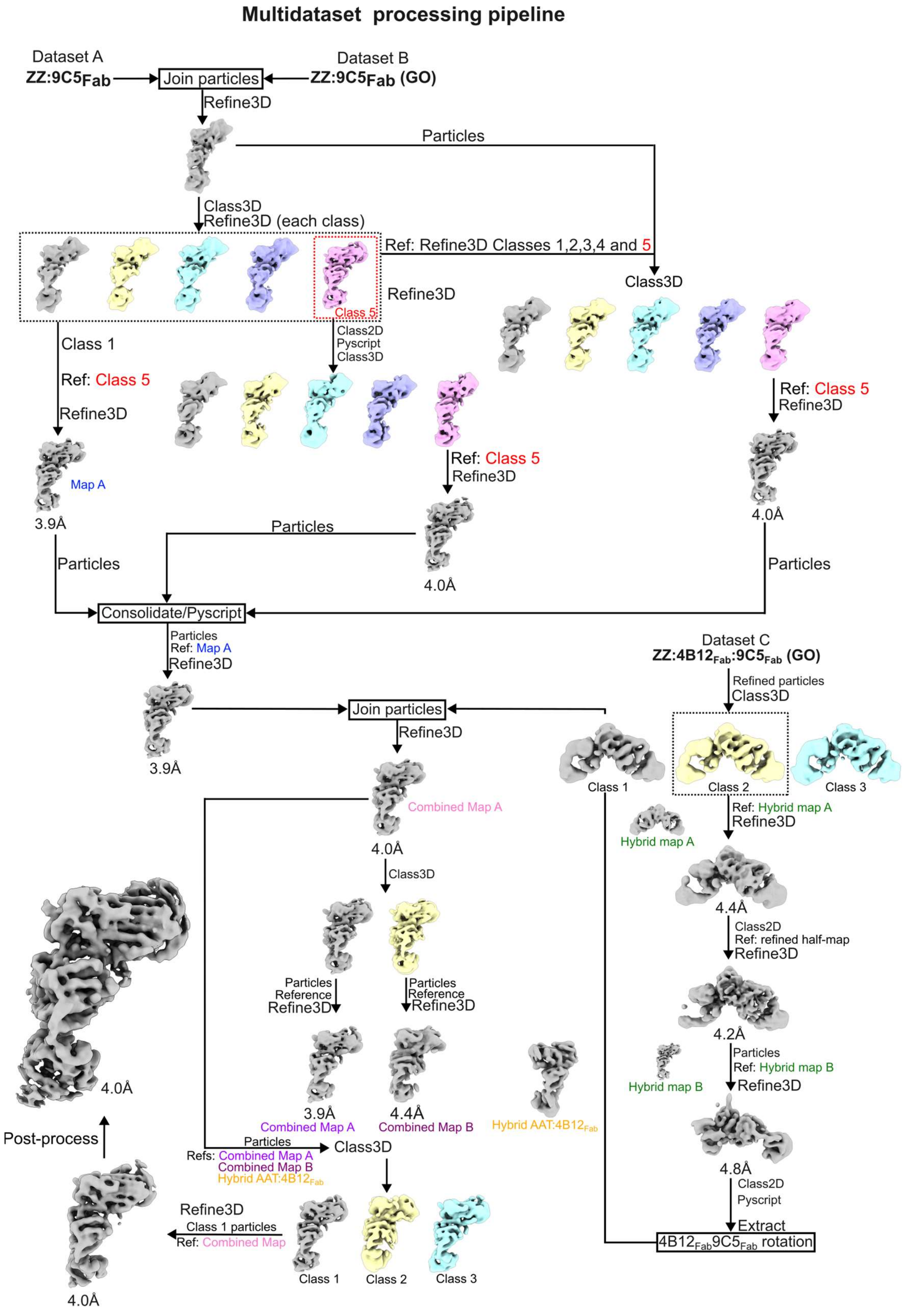

Figure S5. Multi-dataset approach to solve the structure of the Z α_1-antitrypsin polymer subunit with $9C5_{Fab}$. Three datasets of Z α_1-antitrypsin polymers were processed and refined individually, then combined at different stages. Dataset A represents Z α_1-antitrypsin polymers in complex with $9C5_{Fab}$, imaged at 0.93 Å/pixel. Dataset B represents the same sample, vitrified on grids with a graphene oxide (GO) support, at 0.65 Å/pixel. Dataset C represents Z α_1-antitrypsin polymers in complex with both $9C5_{Fab}$ and $4B12_{Fab,}$ imaged at 0.93 Å/pixel. To obtain the

final 4.0 Å map, the refined particles of dataset B were rescaled to match the pixel size of dataset A, and the particle stacks were combined and refined further. Several rounds of 3D classification and refinement were used to identify particles yielding better quality maps of the polymer subunit. Particles related to EM maps with better-resolved density for the α_1-antitrypsin region were progressed and combined with dataset C. Maps that were used as references are given a specific name and color in the diagram.

References

1. A. Dementiev, M. Simonovic, K. Volz, P. G. Gettins, Canonical inhibitor-like interactions explain reactivity of alpha1-proteinase inhibitor Pittsburgh and antithrombin with proteinases. *J Biol Chem* **278**, 37881-37887 (2003).
2. J. A. Huntington, R. J. Read, R. W. Carrell, Structure of a serpin-protease complex shows inhibition by deformation. *Nature* **407**, 923-926 (2000).
3. M. Yamasaki, T. J. Sendall, M. C. Pearce, J. C. Whisstock, J. A. Huntington, Molecular basis of alpha1-antitrypsin deficiency revealed by the structure of a domain-swapped trimer. *EMBO Rep* **12**, 1011-1017 (2011).
4. M. A. Dunstone, W. Dai, J. C. Whisstock, J. Rossjohn, R. N. Pike, S. C. Feil, B. F. Le Bonniec, M. W. Parker, S. P. Bottomley, Cleaved antitrypsin polymers at atomic resolution. *Protein Sci* **9**, 417-420 (2000).
5. T. D. Goddard, C. C. Huang, E. C. Meng, E. F. Pettersen, G. S. Couch, J. H. Morris, T. E. Ferrin, UCSF ChimeraX: Meeting modern challenges in visualization and analysis. *Protein Sci* **27**, 14-25 (2018).
6. S. V. Faull, E. L. Elliston, B. Gooptu, A. M. Jagger, I. Aldobiyan, A. Redzej, M. Badaoui, N. Heyer-Chauhan, S. T. Rashid, G. M. Reynolds, The structural basis for Z α1-antitrypsin polymerization in the liver. *Science Adv* **6**, eabc1370 (2020).
7. N. Motamedi-Shad, A. M. Jagger, M. Liedtke, S. V. Faull, A. S. Nanda, E. Salvadori, J. L. Wort, C. W. Kay, N. Heyer-Chauhan, E. Miranda, An antibody that prevents serpin polymerisation acts by inducing a novel allosteric behaviour. *Biochem J* **473**, 3269-3290 (2016).
8. D. Kimanius, L. Dong, G. Sharov, T. Nakane, S. H. W. Scheres, New tools for automated cryo-EM single-particle analysis in RELION-4.0. *Biochem J* **478**, 4169-4185 (2021).
